# Factors associated with symptom severity in stress-induced exhaustion disorder: cohort characterization and cross-sectional correlations

**DOI:** 10.3389/fpsyt.2025.1548967

**Published:** 2025-06-16

**Authors:** Sean Arthur Cully, Klara Hatinova, Jakob Clason van de Leur, Malin Björnsdotter

**Affiliations:** ^1^ Department of Psychology, University of Gothenburg, Gothenburg, Sweden; ^2^ University of Oxford, Department of Pharmacology/Queen Mary University London, Barts and The London School of Medicine and Dentistry, Gothenburg, Sweden; ^3^ Department of Psychology, Uppsala University, Uppsala, Sweden; ^4^ Department of Psychiatry for Affective Disorders, Sahlgrenska University Hospital, Gothenburg, Sweden; ^5^ Center for Cognitive and Computational Neuroscience (CCNP), Department of Clinical Neuroscience, Karolinska Institutet, Stockholm, Sweden; ^6^ Section of Psychiatry and Neurochemistry, Institute of Neuroscience and Physiology, Sahlgrenska Academy, University of Gothenburg, Gothenburg, Sweden

**Keywords:** exhaustion disorder, burnout, clinical burnout, psychological factors, stress, exhaustion

## Abstract

**Introduction:**

Chronic stress-related conditions such as burnout and exhaustion disorder (ED) constitute a significant and growing individual and societal burden. Still, the long-term interactions between symptoms and key risk factors, including brain structure and function, remain poorly understood. To address this knowledge gap, we initiated the PROMUS project, a large-scale longitudinal brain imaging study of 350 participants on sick leave for ED in Sweden.

**Methods:**

Here, we report baseline cohort (n=300) characteristics and cross-sectional associations between symptom severity, primarily measured using the Shirom-Melamed Burnout Questionnaire (SMBQ), and demographic, occupational, psychiatric, psychological, and lifestyle factors assessed using online questionnaires.

**Results:**

Our findings revealed significant associations between symptom severity and multiple factors, most notably depression, anxiety, sleep disturbances, quality of life, dissociation, psychological inflexibility, intolerance of uncertainty, self-efficacy, alexithymia, trauma, gratitude, educational background, emotional stability, household demands, Attention Deficit/Hyperactivity Disorder symptoms, autistic traits, perfectionism, and physical activity.

**Discussion:**

These findings support previous research linking persistent stress conditions to a spectrum of demographic, occupational, psychiatric, psychological, and lifestyle measures. The results also add to the understanding of targetable ED symptoms and risk factors and set the direction for brain imaging analyses and longitudinal assessments in this cohort.

## Introduction

Sick leave due to stress-induced exhaustion is increasingly prevalent worldwide, contributing to rising healthcare costs, reduced workforce productivity, and a significant decline in overall quality of life (1, 2). In Sweden, chronic stress conditions rival depression in prevalence (3), and are associated with the longest median sickness absence (4) and highest cost burden on the sickness benefits system (3.352bn Swedish krona (SEK) annually) (5). Worryingly, chronic stress is also linked to an array of adverse outcomes, including increased risk of cardiovascular disease, mental health disorders, and cognitive decline (6).

Among the conceptualizations of chronic stress, burnout (7) and exhaustion disorder (ED) are particularly prominent. Burnout originally stems from organizational psychology and aims to describe how an unfavorable relationship between workers and organizational factors may result in symptoms of exhaustion, cynicism, and professional inefficiency (8). Although the burnout construct is widely used and extensively studied, it historically lacked a clear definition and remains a topic of ongoing debate (9). In an effort to bring conceptual clarity, a recent expert consensus defined the concept of burnout as “exhaustion due to prolonged exposure to work-related problems” (10) —a simple and intuitive definition but challenging to operationalize in medical research. Also, this definition targets occupational burnout specifically, suggesting that burnout is caused exclusively by workplace factors. This is consistent with the recent classification of burnout in the International Classification of Diseases, eleventh revision (ICD-11), as an occupational phenomenon, not a medical condition. However, this conceptualization is not well supported by research; in fact, “no clear evidence exists that burnout is primarily caused by work-related stress” (7). In contrast, while also focusing on exhaustion due to chronic stress, ED is a medical diagnosis with specific diagnostic criteria (**Table 1**) and a code in the Swedish International Classification of Diseases (ICD F43.8A) (11). Moreover, ED may be caused by any source of stress, occupational or otherwise (e.g. overwhelming home demands). For the purpose of this study, we therefore consider the general burnout concept to represent a broader, dimensional construct encompassing the chronic stress spectrum, while ED reflects a well-defined clinical entity within this spectrum. Indeed, ED may be considered the end stage of a severe burnout process that requires clinical attention, sometimes also referred to as “clinical burnout” (12). As such, ED offers an internationally unique framework for rigorous medical research in chronic stress, including studies of neurobiological and psychological mechanisms that underlie stress-induced exhaustion. Specifically, ED may serve as a relatively homogenous and well-defined model condition for severe chronic stress within the broader burnout construct. This approach also enables the use of well-established and validated burnout scales to assess stress-related exhaustion symptoms dimensionally within the ED population.

**Table 1:** Exhaustion disorder diagnostic criteria.

All criteria must be fulfilled for an ED diagnosis.
A. Physical and mental symptoms of exhaustion lasting at least 2 weeks. The symptoms have developed in response to one or more identifiable stressors, which have been present for at least 6 months.
B. Marked lack of mental energy, manifested by reduced initiative, lack of endurance, or increased need for recovery following mental efforts.
C. At least four of the following symptoms have been present nearly every day, during the same 2-week period: a. Persistent complaints of impaired memory or concentration. b. Markedly reduced capacity to tolerate demands or to perform under time pressure. c. Emotional instability or irritability. d. Insomnia or hypersomnia. e. Persistent complaints of physical fatigue and lack of endurance. f. Physical symptoms such as muscular pain, chest pain, palpitations, gastrointestinal problems, vertigo, or increased sensitivity to sounds.
D. The symptoms cause clinically significant distress or impairment in social, occupational, or other important areas of functioning.
E. The symptoms are not due to the direct physiological effects of some substance (e.g., drug abuse or medication) or a general medical condition (e.g., hypothyroidism, diabetes, infectious disease).
F. The criteria for major depressive disorder, dysthymic disorder, or generalized anxiety disorder are not met.*

*If the criteria for any of these diagnoses are met ED is to be used as a secondary diagnosis.

Despite the high prevalence and cost, the neurobiology of stress-related exhaustion is severely understudied (13, 14). In their recent reviews, Bayes et al. identified less than ten published brain imaging studies in burnout (13), and Lindsäter et al. identified only five studies in ED (14). While these studies identified alterations in brain regions implicated in stress regulation—such as the prefrontal cortex and amygdala (15–21)—the findings were inconsistent, based on small sample sizes, often in ill-defined populations, and typically lacked control for several potential confounding factors. Also, with the exception of Savic et al. (18), previous research relied exclusively on cross-sectional designs, limiting the ability to examine temporal dynamics or establish causal relationships. Thus, the effect of stress-induced exhaustion on the brain constitutes a major knowledge gap.

To address this gap, we initiated the PROMUS study in which a cohort of 350 participants on sick leave for ED are followed longitudinally using questionnaires covering demographic, occupational, psychiatric, psychological, and lifestyle factors, as well as structural and functional brain imaging. Notably, PROMUS was designed as a dimensional study, recruiting participants from a clinically well-defined population with ED, while using a well-validated burnout scale — the Shirom-Melamed Burnout Questionnaire (SMBQ) — as the primary symptom measure. This approach allows for a nuanced understanding of individual differences in stress-related symptoms and their association with other factors in a relatively homogenous population. At the same time, it facilitates comparison and integration with previous research carried out under the broader and more heterogeneous construct of burnout. Also, a primary objective of the PROMUS study is to ultimately develop predictive models in ED, with 300 participants belonging to the basic cohort subject to extensive data analysis and modeling, while the data from the remaining 50 participants serve as a validation sample that will remain unexamined until final predictive models are generated and can be tested. Currently, there are no such predictive markers or models for burnout or ED (14, 22), although recent findings suggest promising plasma biomarkers (23, 24). A systematic review by Danhof-Pont et al. attributes the lack of consistent biomarkers to the heterogeneity of burnout symptoms and the predominantly cross-sectional design of prior studies, which limits the ability to infer causality (22). The PROMUS study directly addresses these limitations by recruiting participants based on a clinical ED diagnosis, thereby ensuring greater symptom homogeneity.

In the present study, the specific objectives were to (i) conduct a detailed characterization of demographic, occupational, psychiatric, psychological, and lifestyle factors of the 300 participants of the basic PROMUS cohort, including comparisons to the general population, and (ii) identify which individual difference factors are significantly associated with symptom severity, serving as a foundation for the future longitudinal analyses including brain imaging.

The PROMUS study represents a pioneering effort to comprehensively investigate stress-induced exhaustion, a condition with profound individual and societal consequences. By integrating extensive questionnaire data with neuroimaging techniques in a well-defined medical condition, PROMUS aims to elucidate the complex interplay between a broad range of factors associated with chronic stress. Through its longitudinal design and large cohort, we aim to not only deepen our understanding of the neurobiological underpinnings of chronic stress but also develop predictive models that could inform early interventions and personalized treatment strategies. This first report lays the foundation for these goals.

## Materials and methods

### Participants

Participants were recruited via social media ads in the Gothenburg region in Sweden using self-selected convenience sampling. Inclusion criteria were sick leave due to ED and age 18–50 at the time of recruitment. The upper age limit was set to reduce confounding effects of neurobiological changes with older age. Exclusion criteria were brain damage or major neurological disorders (e.g. stroke, multiple sclerosis, etc). Since there is currently no diagnostic ED scale, we did not use any such measure for participant exclusion. As the study was exploratory in nature, no *a priori* power calculation was conducted; instead, the cohort was designed to include the largest feasible sample at a total of 350 participants. Of these, 50 participants were randomly assigned to the validation sample that will remain unexamined until final predictive models are generated, leaving 300 participants in the basic cohort for extensive data analysis.

### Questionnaires

Participants completed an extensive online survey, prepared and distributed electronically through the survey tool esMaker (Entergate). The survey included questions and questionnaires related to demographic, household, occupational, psychiatric, psychological and lifestyle factors.

Demographic and household questions included age, sex (legal gender), gender (self-identified gender), height, weight, years of schooling (including primary, secondary and tertiary formal education), highest degree achieved, number of children, and marital status with the options married, de facto partner living together, de facto partner not living together, partner/girlfriend/boyfriend, and single. Participants were also asked to rate how demanding their household efforts were on a five-point Likert scale ranging from Not demanding at all to Extremely demanding. Body mass index (BMI) was computed from the height and weight information.

For the occupational factors, participants were asked what sector (public or private) and industry they were employed in, their salary level, how many years, months and weeks they had been on sick leave (complete or partial) for ED, and at what percentage and how long they had been on sick leave within the last year. The total number of days on sick leave and the average percentage of sick leave over the past year were then computed. Participants were also asked to rate how physically and mentally demanding their work was. These ratings were made on a five-point Likert scale ranging from Not demanding at all to Extremely demanding.

Symptom severity was primarily assessed with the 22-item Shirom-Melamed Burnout Questionnaire (SMBQ) (25) which is a widely applied measure of chronic stress symptom severity and has been validated in Swedish ED samples (26). We examined the total score as well as each of the four subscales separately: physical exhaustion, cognitive weariness, tension, and listlessness. The physical exhaustion subscale evaluates the extent of an individual’s physical fatigue and depletion of energy. Cognitive weariness measures the degree of cognitive fatigue, including difficulties in concentration and memory impairments. Listlessness assesses a lack of vitality, reflecting disengagement and apathy. Lastly, the tension subscale gauges the level of stress and restlessness experienced by the individual. Collectively, these subscales provide a comprehensive assessment of burnout symptoms, encompassing both physical and psychological dimensions, thus enabling a more detailed understanding of the condition. Approximately halfway into recruitment, the Karolinska Exhaustion Disorder Scale (KEDS), which specifically measures ED symptoms, was added to the questionnaire battery (27). KEDS data is available for *n*=130 participants.

The ED-related psychiatric factors included depression and anxiety (28, 29), current perceived stress levels (30), symptoms of Attention Deficit/Hyperactivity Disorder (ADHD) and autism (31), symptoms of dissociation (32, 33), sleep problems (34), and symptoms of alexithymia (35). In addition, prior traumatic life events (36) and a transdiagnostic measure of cross-cutting psychiatric symptoms were assessed. The 9-item Patient Health Questionnaire (PHQ) was used to assess symptoms of depression (37), the 7-item Generalized Anxiety Disorder Scale (GAD) for levels of anxiety (38), the Perceived Stress Scale (PSS) (39) for stress levels, the 26 item DSM-5 Cross-cutting Symptom Measure (DSM‐XC) (40) as a transdiagnostic measure of cross-cutting psychiatric symptoms, the Adult ADHD Self-report Scale part A (ASRS-A) (41) for ADHD symptoms (scored according to the alternative system with scores ranging from 0 points for a response of “never” to 4 points for “very often”), the Autism-spectrum Quotient questionnaire (AQ) (42) for autistic traits, the 20-item Toronto Alexithymia Scale (TAS) (43) for alexithymic traits, and the PHQ item-3 for sleep problems (44). Past traumatic events were assessed by asking participants if they had witnessed violence, experienced violence, sexual abuse, emotional abuse/bullying, or serious illness or death, and/or other traumatic events. The number of “yes” answers was then computed for a total score (with the maximum score being six).

Participants were also asked to report psychiatric diagnoses other than ED. These reports were grouped into major disorder categories, including variants of the same disorder (e.g. panic disorder, generalized anxiety disorder etc. were grouped into the category anxiety). Stress-related disorders such as adjustment disorders were included in the ED category. Also, we asked participants to report if they had any neurological issues. Finally, we also asked if they had tested positive for Coronavirus disease (COVID-19). Approximately halfway into recruitment a question about how many doses of COVID-19 vaccine they had received was added, and this data is available for 130 participants.

For psychological factors relevant to ED, we included general self-efficacy (45), psychological flexibility (34, 46), perfectionism (34, 47), the Big Five personality traits (including emotional stability, the opposite of neuroticism) (48) and intolerance of uncertainty (49). In addition, we included dispositional gratitude due to its well-established link to general well-being (50) and its apparent causal role in buffering against the negative effects of stress (51, 52). Finally, two psychosocial factors were also assessed: quality of life (53) and social support (54). We assessed Big Five personality dimensions with the Ten Item Personality Inventory (TIPI) (55), general self-efficacy with the 10-item General Self-efficacy Scale (GSES) (56), dissociation symptoms using the Dissociation Screening Questionnaire (DSQ) (57), perfectionism using the 12-item Clinical Perfectionism Questionnaire (CPQ) (58), intolerance of uncertainty with the 12-item Intolerance of Uncertainty Scale-Short Form (IUS) (59), dispositional gratitude with the six-item Gratitude Questionnaire (GQ) (60), psychological inflexibility with the six-item Swedish Acceptance and Action Questionnaire (SAAQ) (61), quality of life with the 12-item Brunnsviken Brief Quality of Life questionnaire (BBQ) (62), and perceived social support with the 12-item Interpersonal Support Evaluation List (ISEL-12) (54).

The included lifestyle factors were alcohol and tobacco use (63), including smoking and snus (a Swedish version of wet snuff used orally), diet (vegetable and snacks consumption) (64), exercise (light and vigorous) (65), and several factors related to family and occupational life (66). Alcohol, tobacco use, exercise and diet were assessed using the questionnaire Levnadsvanor from the Swedish National Board of Health and Welfare. For tobacco use, participants were categorized as having never smoked or used snus, having used it but quit, and using it regularly. For alcohol use, participants were asked to indicate how many drinks they had per week, in groups of 0, 1–4 glasses, 5–9 glasses, 10–14 glasses and more than 14 glasses per week, and how often they drank at least 4 (for women) or 5 (for men) glasses of alcohol including the options never, more rarely than once a month, every month and every day. For exercise, participants were asked how often they participated in vigorous exercise (such as running, gymnastics and soccer) on a 6-point scale ranging from “Never” to “More than 120 minutes per week”, and how often they participated in light exercise (such as walking, biking or gardening) for at least 10 minutes, on a 7-point scale ranging from “Less than 30 minutes per week” to “More than 300 minutes (5 hours) per week”. Finally, participants were asked how often they consumed vegetables and snacks (such as cookies, chocolate, candy and soft drinks) on a 4-point scale ranging from “Once a week or less” to “Twice or more per day”.

### Data analysis

The reliability of the questionnaire results was assessed with Cronbach’s alpha, and common method bias was assessed using Harman’s single-factor test.

Descriptive data were summarized for each measure and compared to norms, previous results in general populations, and cut-off scores where available as indicated. For personality traits, two-tailed t-tests were used to assess significant group differences between the ED sample and norm data (67) for each sex and age group and the resulting *p*-values were adjusted for multiple comparisons using Benjamini-Hochberg false discovery rate (FDR (68); at a significance threshold α = .05). For GSES scores, individual mean scores (i.e. the total score divided by the number of questions) were used for comparison with the Swedish data from a randomized sample (56). Descriptive data analyses were conducted in Matlab R2022b (MathWorks, Natick, MA, USA).

Correlation tests were used to assess associations between burnout symptoms, quantified as total SMBQ scores as well as each of the subscales, i.e. physical exhaustion, cognitive weariness, tension and listlessness, and the other variables of interest. The nonparametric Spearman’s rho was used as several variables were ordinal or had nonnormal distributions. One-tailed tests were used for all analyses with hypothesized directional associations. The *p*-values were adjusted for multiple comparisons using the FDR approach, and assessed at a significance threshold of α = .05. Correlation analyses were conducted in R (R Core Team, 2022). For comparison, the same approach was also used to assess associations between the factors and the KEDS scores in participants who completed this scale.

For the demographic factors, we hypothesized that burnout symptoms would be positively associated with BMI (69), number of children (70) and household demand level (71), and negatively associated with years of schooling (72). For the occupational factors, we hypothesized that burnout symptoms would be positively associated with sick leave in the past year and total sick leave, physical demand level and mental demand level (73), and negatively associated with salary level (74). For psychiatric factors, we hypothesized that burnout symptoms would be positively associated with all psychiatric measures (28, 29, 31–36). For the psychological factors, we hypothesized that burnout symptoms would be positively associated with psychological inflexibility (34, 46), perfectionism (34, 47), and intolerance of uncertainty (49), and negatively associated with general self-efficacy (45, 75), dispositional gratitude (50–52), all Big Five personality traits (openness to experiences, conscientiousness, extraversion, and agreeableness, and emotional stability) (48, 75), quality of life (53), and social support (76). Finally, for the lifestyle factors, we hypothesized that burnout symptoms would be positively associated with alcohol use (63), nicotine use (63), and snacks consumption (64), and negatively associated with vegetable consumption (64), and light as well as vigorous exercise frequency (65).

## Results

### Questionnaire data

Cronbach’s alpha for the TIPI was 0.66, indicating modest internal consistency aligned with previous findings and the TIPI design (55), 0.71 for BBQ and between 0.80 and 0.93 for the remaining measures. Harman’s single-factor test results showed that the questionnaire variance was 15%, suggesting low common method bias.

### Participant characteristics

**Figure 1:**
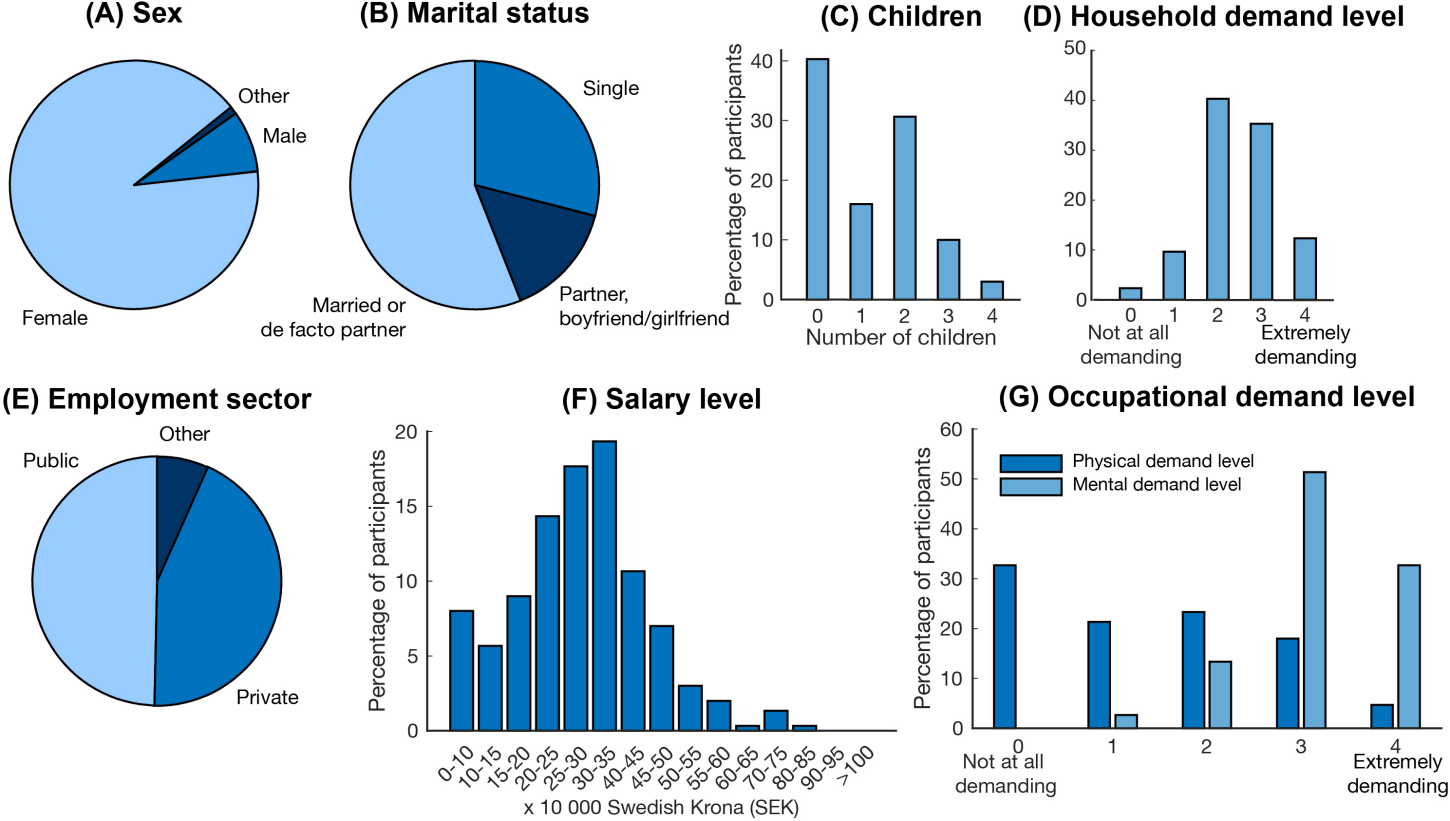
Demographic and occupational characteristics of the cohort. **(A)** sex, **(B)** marital status, **(C)** number of children, **(D)** level of household demand, **(E)** employment sector, **(F)** salary level, and **(G)** level of occupational demand.

The majority (91%) of the participants were women and the average age was 38 years (ranging from 23 years to 49 years old) (**Table 2**, **Figure 1A**). The majority were married or de facto partners living together (56%), while 15% had a partner (e.g. boyfriend or girlfriend) and 29% were single (**Figure 1B**). 60% of participants had at least one child, while 40% did not have children (**Figure 1C**). Participants’ mean BMI was 26.38 (std = 5.43), with 48% in the healthy weight range and 51% in the overweight or obese range. The average years of schooling were 15 years (std = 2.95 years), and most participants (76%) had obtained a university-level or corresponding degree after secondary school. Four participants reported years of schooling of less than 9 years, which is the mandatory number of years in Sweden, suggesting that they may have misunderstood the question. The household demand level was reported as moderately (40%) or very (35%) demanding by the majority of the participants (**Figure 1D**).

**Table 2:** Participant characteristics.

Demographic measures		
Sex, female:male:other	273:24:03	
Gender, women:men:other	264:24:12	
	Mean (sth)	Median [Min-Max]
Age	38.01 (5.50)	39 [23-49]
Number of children	1.19 (1.16)	1 [0-4]
Schooling (years)	15.41 (2.96)	16 [3-24]
BMI	26.38 (5.43)	25.13 [17.10-47.61]
Occupational measures		
Total lifelong sick leave, days	933 (1071)	462 [0-6205]
Sick leave in the past year (%)	63.94 (32.03)	62.50 [3.57-100]
Psychiatric measures		
SMBQ	5.42 (0.79)	5.50 [2.59-6.91]
SMBQ-Cognitive fatigue	5.55 (1.04)	5.83 [2-7]
SMBQ-Physical exhaustion	5.47 (0.97)	5.63 [2.50-7]
SMBQ-Listlessness	5.53 (0.89)	5.50 [2.25-7]
SMBQ-Tension	4.99 (1.17)	5 [2-7]
KEDS	33.72 (8.4)	33 [9-52]
PSS	30.51 (2.91)	31 [23-38]
PHQ	13.2 (5.46)	13 [0-27]
PHQ Sleep	1.96 (1.06)	2 [0-3]
GAD	8.84 (5.00)	8 [0-21]
AQ	18.32 (9.65)	16 [1-44]
ASRS-A	12.77 (5.40)	12.50 [1-24]
TAS	47.72 (13.25)	46 [20-78]
Trauma	3.29 (1.95)	4 [0-6]
DSM‐XC	35.13 (13.13)	33.50 [7-78]
Psychological measures		
BBQ	38.73 (18.41)	37 [0-96]
ISEL	23.75 (7.71)	25 [0-36]
CPQ	34.48 (6.05)	36 [13-46]
DSQ	13.55 (8.42)	12 [0-44]
IUS	34.71 (9.87)	35 [13-60]
GQ	32.85 (7.17)	34 [12-42]
GSES	27.61 (5.51)	28 [10-40]
SAAQ	27.52 (9.33)	28 [7-49]
TIPI Extraversion	4.74 (1.54)	5 [1-7]
TIPI Agreeableness	5.55 (1.14)	6 [1-7]
TIPI Conscientiousness	4.86 (1.42)	5 [1-7]
TIPI Emotional Stability	4 (1.45)	4 [1-7]
TIPI Openness	5.43 (1.13)	5.50 [2-7]

AQ, Autism-spectrum Quotient; ASRS, Adult ADHD Self-report Scale; BBQ, Brunnsviken Brief Quality of Life; BMI, Body mass index; CPQ, Clinical Perfectionism Questionnaire; DSM‐XC, DSM-5 Cross-cutting Symptom Measure; DSQ, Dissociation Screening Questionnaire; GAD, Generalized Anxiety Disorder Scale; GQ, Gratitude Questionnaire; GSES, General Self-efficacy Scale; ISEL, Interpersonal Support Evaluation List; IUS, Intolerance of Uncertainty Scale; KEDS, Karolinska Exhaustion Disorder Scale; PHQ, Patient Health Questionnaire; PSS, Perceived Stress Scale; SAAQ, Swedish Acceptance and Action Questionnaire; SMBQ, Shirom-Melamed Burnout Questionnaire; TAS, Toronto Alexithymia Scale; TIPI, Ten Item Personality Inventory.

44% of the participants reported employment in the private sector, and 50% in the public sector (**Figure 1E**). The majority worked in a medical (26%), pedagogical (11%), social (9%) or administrative (9%) type of employment. The most common profession was nurse (16%), followed by teacher (11%) and social worker (4%); however, a wide range of professions were represented in the sample. The majority of the participants (51%) had a pre-tax salary of 20-35–000 SEK per month (1,900 - 3,300 United States Dollar), with a span from 0-10 000 SEK up to 70-75 000 SEK per month (**Figure 1F**). Most participants, 77%, reported that their occupation was “not at all” or “a little bit” physically demanding, while 51% reported that their occupation was extremely mentally demanding (**Figure 1G**).

**Figure 2:**
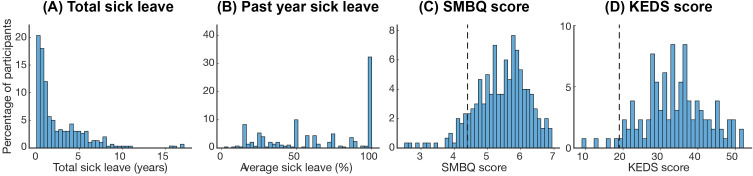
Sick leave and exhaustion disorder severity. Histograms showing **(A)** years on sick leave, **(B)** average percent sick leave in the past year, **(C)** SMBQ scores, and **(D)** KEDS scores. Dotted lines in panels **(C, D)** indicate the cut-off points for the respective scale. SMBQ, Shirom-Melamed Burnout Questionnaire; KEDS, Karolinska Exhaustion Disorder Scale.

For long-term sick leave, measured as the total days on sick leave due to ED, 27 participants reported 0. When these participants were excluded, the average sick leave was 1025 days (2.90 years). However, the span was very large, ranging from one month to 17.5 years (**Figure 2A**). The majority had been on sick leave for 1–2 years (52%), while fewer had been on leave for 2–3 years (7%), 3–4 years (8%), 4–5 years (8%), 5–10 years (14%) or more than 10 years (2%). The average percentage of sick leave in the past year was 64%, ranging from 4% to 100%, with 32% of the participants reporting full-time (100%) sick leave (**Figure 2B**). The average SMBQ score was 5.4, and 270 (90%) participants scored above the 4.4-point SMBQ cut-off for severe burnout (26) (**Figure 2C**). Participants who reported employment in the public sector did not differ from those employed in the private sector in SMBQ scores, the average percentage of sick leave in the past year, or the total number of sick leave days (all *p*>0.3). Of the 130 participants with KEDS data, 96% scored above the 19-point cutoff for ED (27) (**Figure 2D**).

41% of the participants reported at least one occasion of COVID-19. There were no significant group differences in SMBQ score or average sick leave in the past year between participants who had been infected compared to those who had not, and there were no significant correlations between SMBQ scores or average sick leave in the past year and number of doses of COVID-19 vaccine (all *p*>0.3).

**Figure 3:**
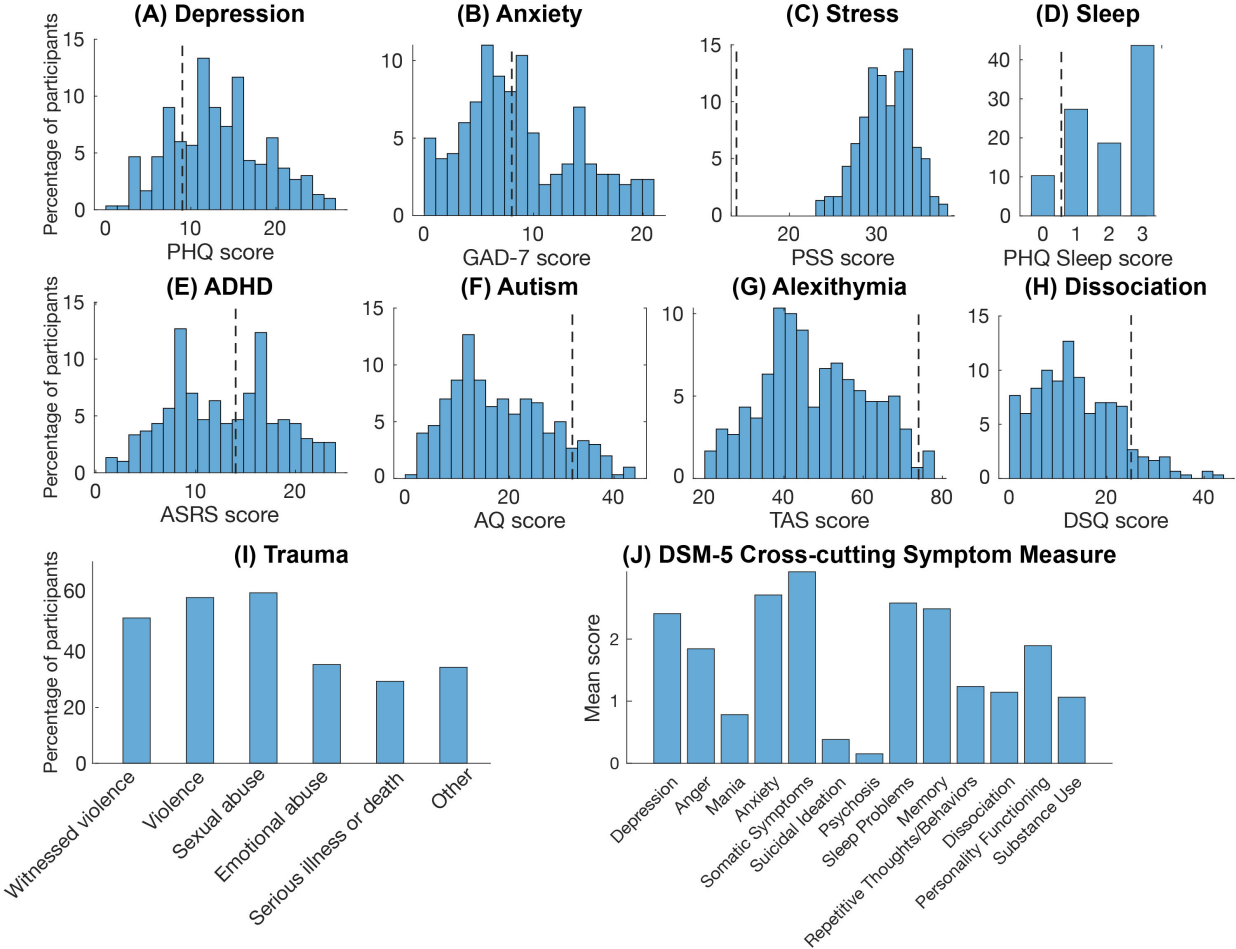
Psychiatric measures. Histograms showing participants scores on the psychiatric measures. **(A)** depression symptoms **(B)** anxiety symptoms, **(C)** perceived stress level, **(D)** sleep problems, **(E)** ADHD symptoms, **(F)** autistic traits, **(G)** alexithymic traits, **(H)** dissociative symptoms, **(I)** past trauma experiences, and **(J)** Cross-cutting DSM-5 psychiatric symptoms. Dotted lines indicate the cut-off points for the respective scales where available. PHQ, Patient Health Questionnaire; GAD-7, Generalized Anxiety Disorder Scale; PSS, Perceived Stress Scale; ASRS, Adult ADHD Self-report Scale; AQ, Autism-spectrum Quotient; TAS, Toronto Alexithymia Scale; DSQ, Dissociation Screening.

The majority of participants (68%) reported comorbidity with at least one other psychiatric disorder. The most common were depression (41%), anxiety (23%), ADHD (10%), autism (4%), post-traumatic stress disorder (PTSD) (8%), borderline personality disorder (2%), obsessive-compulsive disorder (OCD) (2%), eating disorders (2%) and bipolar disorder (2%). 16% of participants reported both depression and anxiety, while 32% reported no comorbid psychiatric disorder. Consistently, 73% of the participants had a PHQ score greater than or equal to 10, which is indicative of major depression (77) (**Figure 3A**), and 36% had a GAD score of 10 or greater indicative of moderate to severe anxiety; however, only 15% scored above the 15 point cutoff for severe anxiety (38) (**Figure 3B**). For the PSS scale, there are no formal cutoff points; however, the latest established Swedish norm is a mean score of 13.97 (std = 6.34) compared to which the participants scored substantially higher with a mean score of 30.51 (std = 2.91) (30) (**Figure 3C**). For sleep problems, 90% of the participants scored at least 1 on the PHQ sleep item (item 3) which is indicative of sleep disturbance (44) (**Figure 3D**). Surprisingly, 46% had ASRS-A scores of 14 or above which is indicative of ADHD (41) (**Figure 3E**) while 10% had AQ scores above 32 (**Figure 3F**), indicating clinically significant levels of autistic traits (42). Also, only 2% had a TAS score equal to or greater than 74 – values above this indicate alexithymia (78) (**Figure 3G**). 10% reported a DSQ score above 25 points, the cutoff for dissociation (57) (**Figure 3H**). A large proportion of the participants had experienced traumatic events, ranging from 29% for serious illness or death to 61% for sexual abuse (**Figure 3I**). Consistent with the self-reported diagnoses and the questionnaire results, the DSM‐XC scores indicated that participants had relatively high levels of depression, anxiety and sleep problems, but also high levels of somatic symptoms, memory problems, anger, and personality functioning. However, scores were low on mania, suicidal ideation, psychosis, repetitive thoughts and behaviors, dissociation, and substance use (**Figure 3J**).

The majority (83%) reported no neurological conditions, and the remaining reports were of minor conditions such as previous concussions (3%) and migraine (4%). A small number also reported chronic pain conditions (1%). In addition, three participants reported myalgic encephalomyelitis and/or chronic fatigue syndrome (ME/CFS).

57% of the participants reported that they exercised vigorously less than 30 minutes per week or more rarely. However, 65% reported that they moved, for example by walking or biking, at least 90–150 minutes per week or more. Also, 71% reported that they consumed vegetables a few times per day or more, and 78% reported consuming snacks or soda a few times per week or more. 66% had never smoked, 27% had quit smoking, and 8% smoked regularly. 78% had never used snus, 7% had quit snus, and 15% used snus regularly. 87% reported that they never or rarely drank more than 4 or 5 glasses of alcohol, for women and men respectively, while 10% reported drinking that much every month and 3% every week. 64% reported drinking less than one glass of alcohol per week, while 28% reported drinking 1–4 glasses per week and 8% reported 5–9 glasses per week. No one reported drinking more than 9 glasses per week.

**Figure 4:**
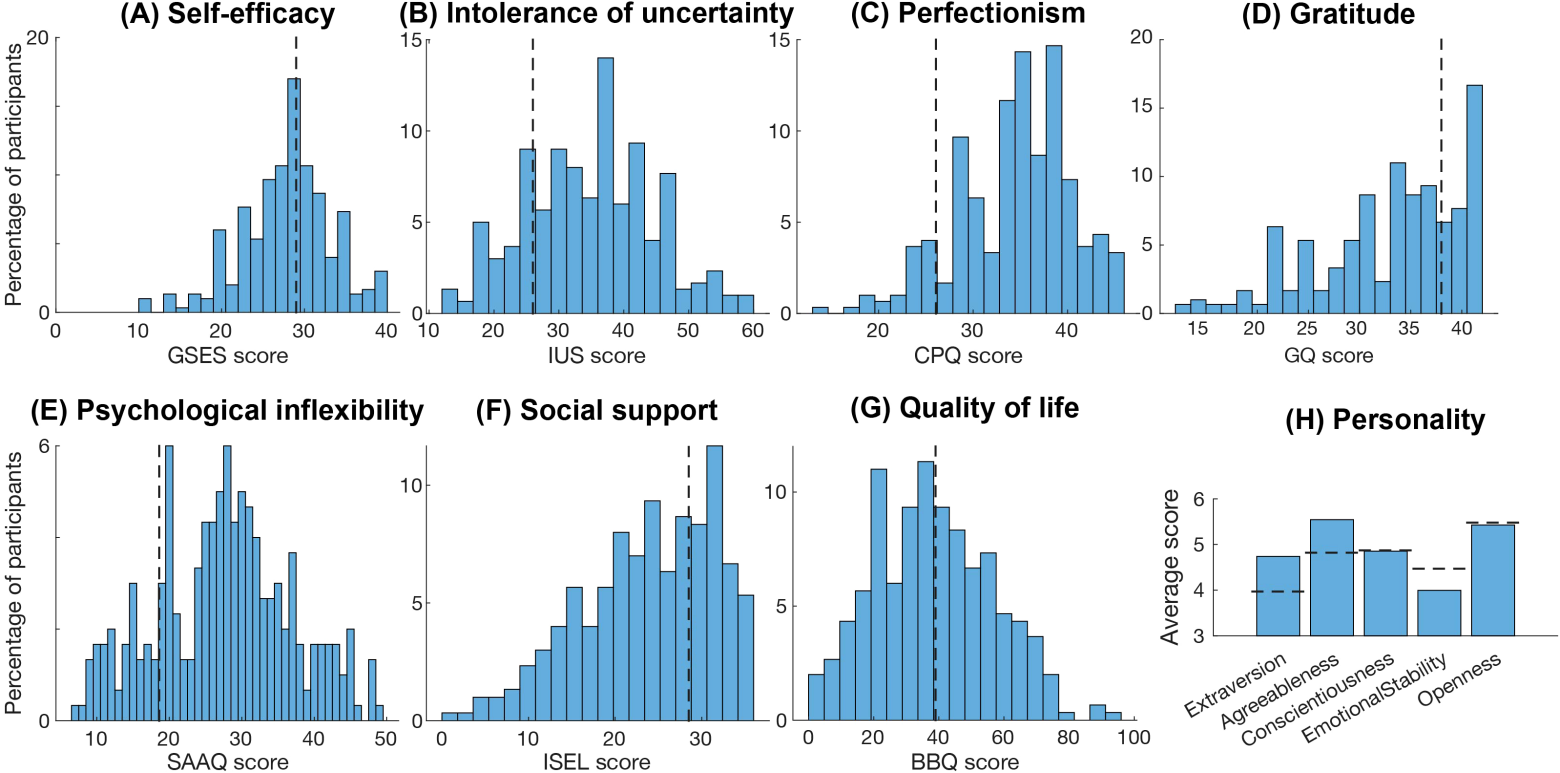
Psychological measures. Histograms showing participants scores on the psychological measures. **(A)** perceived self-efficacy, **(B)** intolerance of uncertainty, **(C)** perfectionism, **(D)** proneness to experience gratitude, **(E)** level of psychological inflexibility, **(F)** perceived social support, **(G)** quality of life, and **(H)** Big Five personality traits. Dotted lines indicate reference scores compared to non-clinical groups, norm data or cut-offs for the respective scales where available. GSES, General Self-efficacy Scale; IUS, Intolerance of Uncertainty Scale; CPQ, Clinical Perfectionism Questionnaire; GQ, Gratitude Questionnaire; SAAQ, Swedish Acceptance and Action Questionnaire; ISEL, Interpersonal Support Evaluation List; BBQ, Brunnsviken Brief Quality of Life.

For psychological factors, women had significantly lower general self-efficacy (GSES scores) than a reference sample (mean = 2.74, std = 0.55 compared to mean = 2.90, std = 0.47; *p*<0.001) (**Figure 4A**) (56), but there was no significant difference in scores for men (mean = 3.00, std = 0.58 compared to mean = 3.03, std = 0.45; *p*=0.75). Participants also had higher intolerance of uncertainty with significantly higher IUS scores (mean = 34.71, std = 9.87) compared to a reference sample (mean = 25.85, std = 9.45; *p*<0.001) (79) (**Figure 4B**). For perfectionism, participants had significantly higher CPQ scores (mean = 34.48, std = 6.05) compared to a non-clinical reference sample (mean = 26.53, std = 4.76) (80) but significantly lower scores than a sample self-selected for severely problematic perfectionism (mean = 38.30, std = 4.60, *p*<0.001) (81) (**Figure 4C**). For gratitude, the ED participants did not significantly differ in GQ scores (mean = 32.85, std = 7.17) compared to a reference sample (mean = 32.51, std = 5.14) (82) (**Figure 4D**). For psychological inflexibility, the study participants had substantially and significantly higher SAAQ scores (i.e. higher psychological inflexibility) (mean = 27.52, std = 9.33) than a reference sample (mean = 18.71, std = 7.7; *p*<0.001) (**Figure 4E**). However, the SAAQ scores did not significantly differ from those in that sample that reported major depressive disorder (mean = 26.70, std = 9.55; *p* = 0.704). For social support, the participants had similar ISEL scores (mean = 29.76, std = 7.1) to a reference sample (mean = 28.57, std = 5.79, *p*=0.091) (83) (**Figure 4F**). 52% reported a BBQ score below 39, the cutoff for unspecific but notable psychopathology (84) (**Figure 4G**).

For personality traits, we found significant associations in specific age groups (**Table 3**). Women in all age groups scored significantly higher on agreeableness compared to norm data. For extraversion, women aged 31–50 and men aged 21–30 scored significantly higher than the norm (**Table 3**) (67). All women’s scores for emotional stability were below the norm, but this difference only reached significance in the 31–40 age group. For conscientiousness and openness, there were no consistent findings across the age groups. It should be noted, however, that the number of male participants was small and the results in the male groups should be interpreted with caution. Moreover, the Cronbach’s alfa (0.66) suggests a modest internal consistency and these results should be interpreted with caution.

**Table 3:** Ten item personality measure (TIPI) results.

Measure	Female sample	Female norm	*t*-test *p*-value	Male sample	Male norm	*t*-test *p*-value
Extraversion	mean (std)	mean (std)	*p*	mean (std)	mean (std)	*p*
21-30	4.39 (1.30)	4.07 (1.61)	0.564	5.60 (1.40)	3.73 (1.54)	0.030
31-40	4.80 (1.50)	4.17 (1.64)	0.000	4.20 (1.30)	3.81 (1.54)	0.547
41-50	4.80 (1.60)	4.20 (1.64)	0.000	4.10 (1.70)	3.85 (1.54)	0.625
Agreeableness						
21-30	5.90 (0.88)	4.88 (1.19)	0.000	5.20 (0.91)	4.50 (1.20)	0.339
31-40	5.50 (1.30)	5.04 (1.19)	0.000	5.40 (0.89)	4.55 (1.21)	0.067
41-50	5.60 (1.10)	5.28 (1.17)	0.025	5.21 (1.00)	4.70 (1.18)	0.365
Conscientiousness						
21-30	4.80 (1.50)	4.78 (1.41)	0.939	5.20 (1.40)	4.57 (1.39)	0.467
31-40	4.70 (1.40)	4.97 (1.41)	0.079	4.10 (1.50)	4.77 (1.35)	0.214
41-50	5.10 (1.40)	5.18 (1.36)	0.625	4.60 (1.80)	4.96 (1.35)	0.564
Emotional Stability						
21-30	3.50 (1.50)	4.09 (1.45)	0.079	4.70 (1.00)	4.64 (1.46)	0.939
31-40	3.90 (1.50)	4.25 (1.45)	0.025	3.60 (1.30)	4.63 (1.42)	0.060
41-50	4.30 (1.40)	4.49 (1.45)	0.326	3.90 (1.60)	4.72 (1.39)	0.214
Openness						
21-30	5.20 (0.96)	5.55 (1.12)	0.214	6.20 (0.84)	5.49 (1.13)	0.320
31-40	5.40 (1.20)	5.49 (1.18)	0.540	5.90 (0.81)	5.49 (1.12)	0.365
41-50	5.40 (1.20)	5.46 (1.20)	0.671	5.50 (1.30)	5.41 (1.17)	0.887

Two-tailed *t*-tests with FDR-corrected *p*-values. Sample sizes for the age groups 21-30:31-40:41:50 were 29:135:109 for females and 5:11:8 for males.

### Correlation analyses

We found several statistically significant correlations between the level of burnout symptoms (*p*<.05, FDR adjusted for multiple comparisons) (**Table 4**). All correlation results are reported in **Supplementary Table 1**.

**Table 4:** Significant correlations with SMBQ scores.

Category	Measure	SMBQ total		Cognitive fatigue		Physical exhaustion		Listlessness		Tension	
		ρ	*p*	ρ	*p*	ρ	*p*	ρ	*p*	ρ	*p*
*Demographic*	Schooling	-0.22	0.000	-0.22	0.000	-0.15	0.008	-0.13	0.033	-0.15	0.009
	Household demand level	0.21	0.000	0.21	0.001	0.19	0.001	0.09	0.134	0.17	0.003
	BMI	0.17	0.002	0.18	0.002	0.14	0.010	0.09	0.111	0.07	0.173
	Number of children	0.09	0.090	0.16	0.006	0.10	0.058	0.00	0.639	-0.02	0.697
	Salary level	-0.03	0.328	0.02	0.667	-0.06	0.205	0.08	0.915	-0.13	0.021
*Lifestyle*	Vigorous exercise	-0.24	0.000	-0.10	0.063	-0.29	0.000	-0.26	0.000	-0.01	0.498
	Light exercise	-0.19	0.001	-0.13	0.026	-0.20	0.001	-0.22	0.000	0.01	0.646
	Snack consumption	0.13	0.019	0.04	0.293	0.12	0.029	0.20	0.001	0.08	0.142
	Smoking	0.03	0.328	0.03	0.416	0.02	0.424	-0.02	0.734	0.11	0.045
*Occupational*	Sick leave past year (%)	0.18	0.002	0.16	0.008	0.15	0.008	0.10	0.086	0.10	0.073
	Physical demand at work	0.14	0.010	0.14	0.013	0.12	0.032	0.00	0.631	0.16	0.007
*Psychiatric*	PHQ	0.62	0.000	0.48	0.000	0.57	0.000	0.36	0.000	0.42	0.000
	DSM‐XC	0.56	0.000	0.40	0.000	0.47	0.000	0.20	0.001	0.56	0.000
	GAD	0.55	0.000	0.39	0.000	0.47	0.000	0.21	0.001	0.59	0.000
	Sleep problems	0.41	0.000	0.29	0.000	0.45	0.000	0.26	0.000	0.17	0.003
	DSQ	0.35	0.000	0.31	0.000	0.27	0.000	0.12	0.041	0.38	0.000
	PSS	0.26	0.000	0.20	0.001	0.17	0.004	0.14	0.028	0.26	0.000
	TAS	0.26	0.000	0.22	0.000	0.15	0.008	0.19	0.001	0.25	0.000
	Trauma	0.24	0.000	0.17	0.004	0.24	0.000	0.10	0.079	0.16	0.007
	ASRS-A	0.21	0.000	0.19	0.002	0.16	0.005	0.04	0.345	0.25	0.000
	AQ	0.20	0.001	0.13	0.022	0.15	0.008	0.08	0.155	0.23	0.000
*Psychological*	SAAQ	0.35	0.000	0.20	0.001	0.28	0.000	0.24	0.000	0.38	0.000
	IUS	0.33	0.000	0.22	0.000	0.26	0.000	0.14	0.019	0.34	0.000
	CPQ	0.20	0.001	0.06	0.219	0.21	0.000	0.07	0.195	0.22	0.000
	ISEL	-0.14	0.012	-0.10	0.067	-0.15	0.008	-0.11	0.063	-0.05	0.250
	Emotional stability	-0.22	0.000	-0.11	0.060	-0.17	0.004	-0.08	0.165	-0.32	0.000
	GQ	-0.23	0.000	-0.12	0.036	-0.23	0.000	-0.17	0.005	-0.16	0.007
	GSES	-0.27	0.000	-0.19	0.001	-0.21	0.000	-0.18	0.004	-0.21	0.000
	BBQ	-0.38	0.000	-0.28	0.000	-0.33	0.000	-0.26	0.000	-0.27	0.000

One-tailed correlation tests with FDR-adjusted *p*-values.

AQ, Autism-spectrum Quotient; ASRS-A, Adult ADHD Self-report Scale part A; BBQ, Brunnsviken Brief Quality of Life; BMI, Body mass index; CPQ, Clinical Perfectionism Questionnaire; DSQ, Dissociation Screening Questionnaire; GAD, Generalized Anxiety Disorder Scale; GQ, Gratitude Questionnaire; GSES, General Self-efficacy Scale; HAD, Hospital Anxiety and Depression Scale; ISEL, Interpersonal Support Evaluation List; IUS, Intolerance of Uncertainty Scale; KEDS, Karolinska Exhaustion Disorder Scale; PHQ, Patient Health Questionnaire; PSS, Perceived Stress Scale; SAAQ, Swedish Acceptance and Action Questionnaire; SMBQ, Shirom-Melamed Burnout Questionnaire; TAS, Toronto Alexithymia Scale.

**Figure 5:**
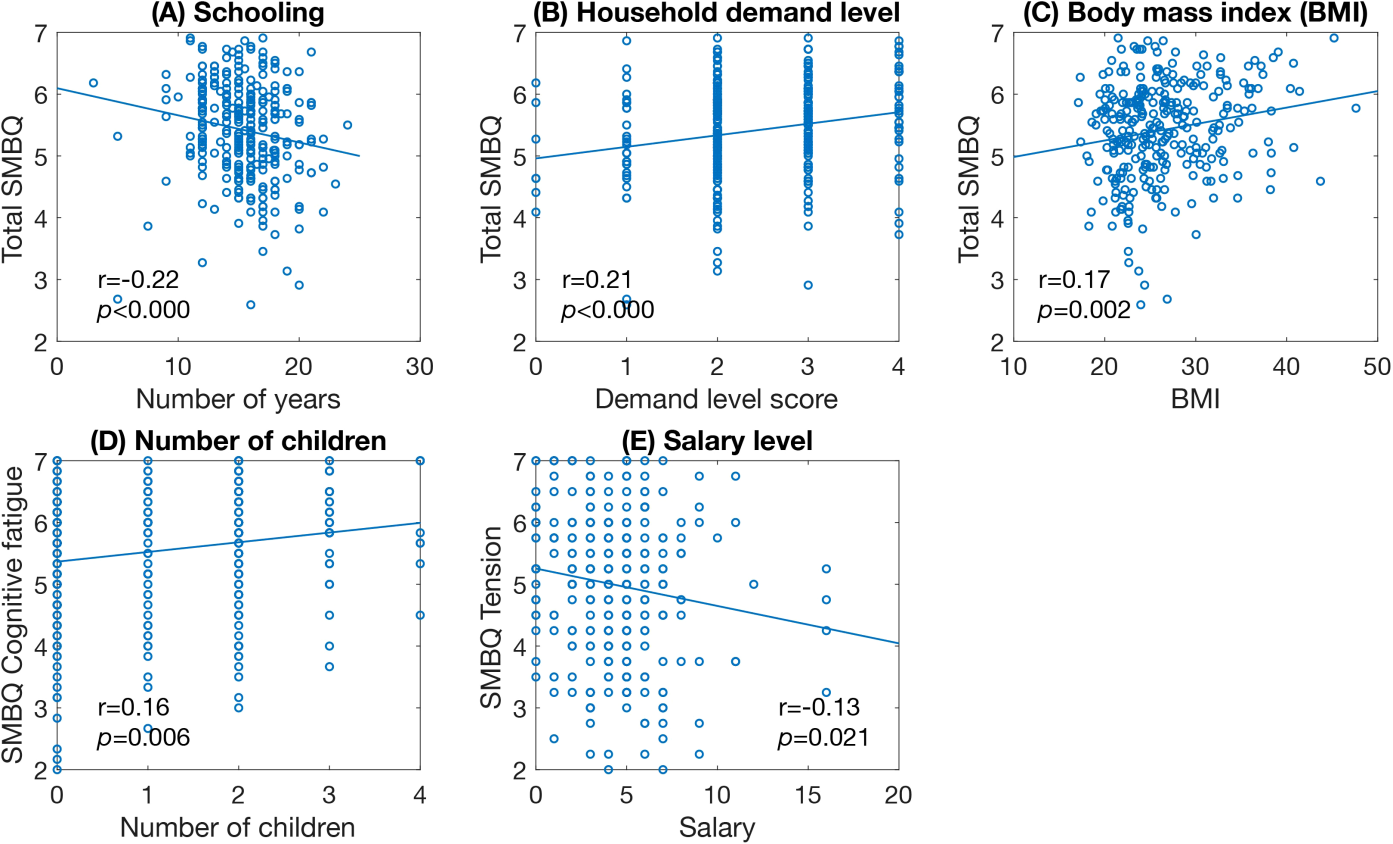
Scatter plots of significant correlations between SMBQ scores and demographic factors. **(A)** years in school, **(B)** level of household demand, **(C)** body mass index, **(D)** number of children, and **(E)** salary level. Scatter plots showing the significant correlations between total SMBQ scores and participants scores on the demographic measures. The *p*-values are false discovery rate (FDR) adjusted for multiple comparisons. SMBQ, Shirom-Melamed Burnout Questionnaire, BMI, Body mass index.

Of the demographic factors, years of schooling was significantly correlated with the total SMBQ score (**Figure 5A**) and each of the subscales (**Table 4**). The association became stronger when the four participants who reported less than 9 years of schooling were excluded. Household demand level was significantly correlated with total SMBQ scores (**Figure 5B**), cognitive fatigue, physical exhaustion, and tension. BMI was significantly correlated with SMBQ total score (**Figure 5C**), cognitive fatigue, and physical exhaustion. Additionally, the number of children was correlated with cognitive fatigue, and salary level was correlated with tension (**Figures 5D, E**).

**Figure 6:**
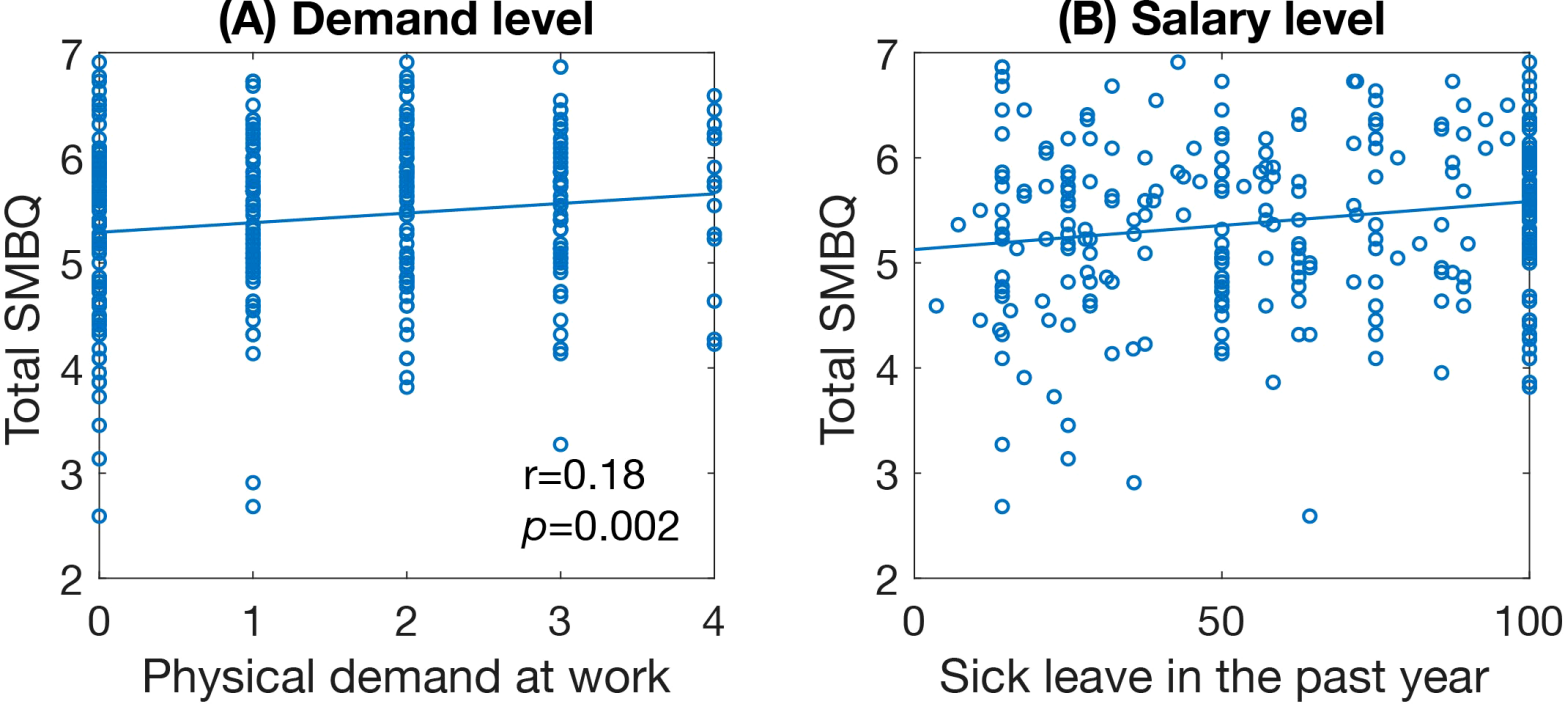
Scatter plots of significant correlations between SMBQ scores and occupational factors. **(A)** level of physical occupational demand and **(B)** salary level. Scatter plots showing the significant correlations between total SMBQ scores and participants scores on the occupational measures. The *p*-values are false discovery rate (FDR) adjusted for multiple comparisons. SMBQ, Shirom-Melamed Burnout Questionnaire.

Of the occupational factors, physical demand at work correlated significantly with total SMBQ scores (**Figure 6A**) and all SMBQ subscales except listlessness. Sick leave in the past year was significantly correlated with total SMBQ score (**Figure 6B**), cognitive fatigue, and physical exhaustion.

**Figure 7:**
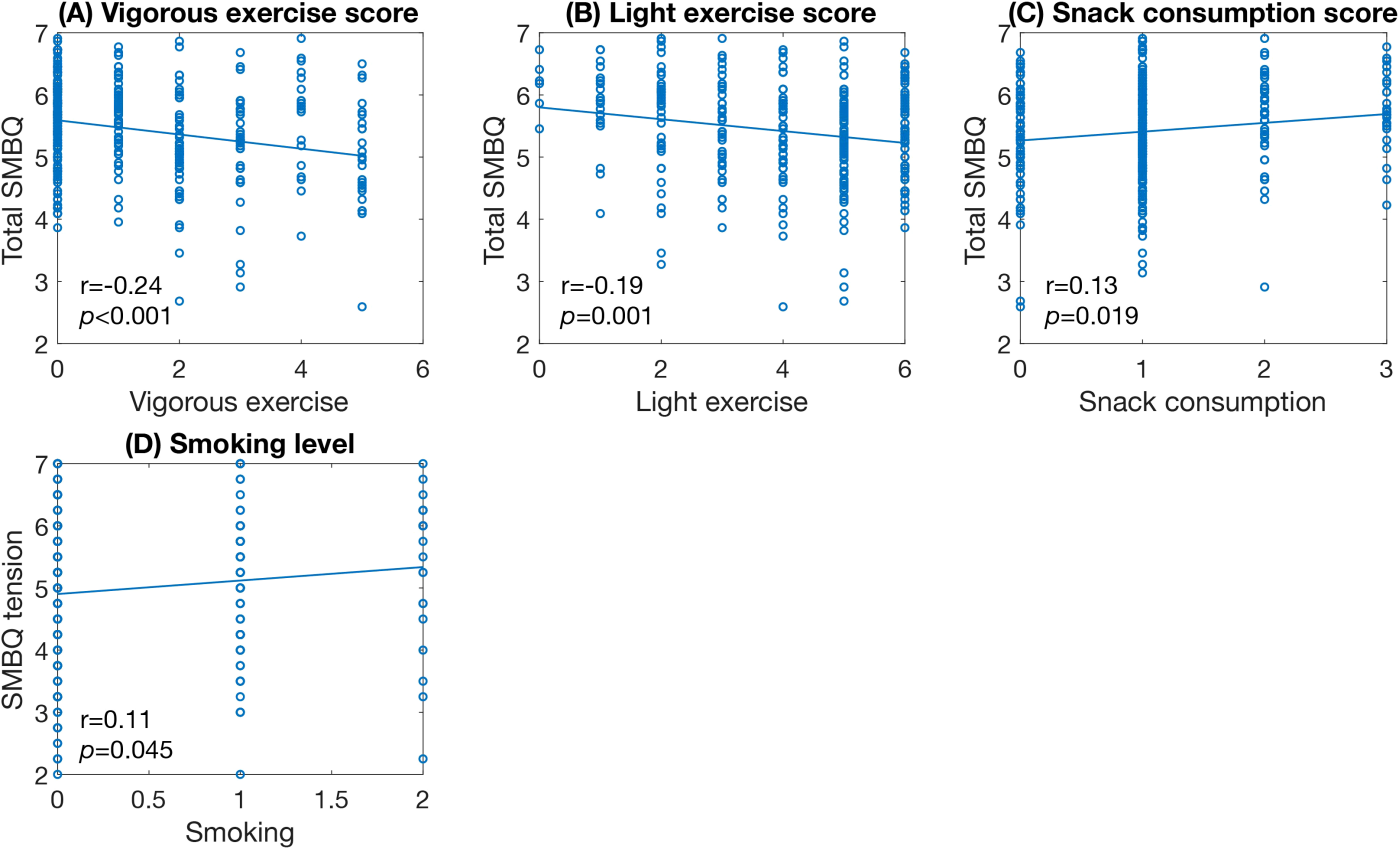
Scatter plots of significant correlations between SMBQ scores and lifestyle factors. **(A)** amount of vigorous exercise, **(B)** amount of light exercise, **(C)** frequency of snack consumption, and **(D)** amount of smoking. Scatter plots showing the significant correlations between total SMBQ scores and participants scores on the lifestyle measures. The *p*-values are false discovery rate (FDR) adjusted for multiple comparisons. SMBQ, Shirom-Melamed Burnout Questionnaire.

For lifestyle factors, both vigorous and light exercise frequency correlated with total SMBQ scores (**Figures 7A, B**) and all SMBQ subscales except tension. Snack consumption correlated significantly with total SMBQ score (**Figure 7C**), physical exhaustion, and listlessness. Smoking was only significantly correlated with tension (**Figure 7D**).

**Figure 8:**
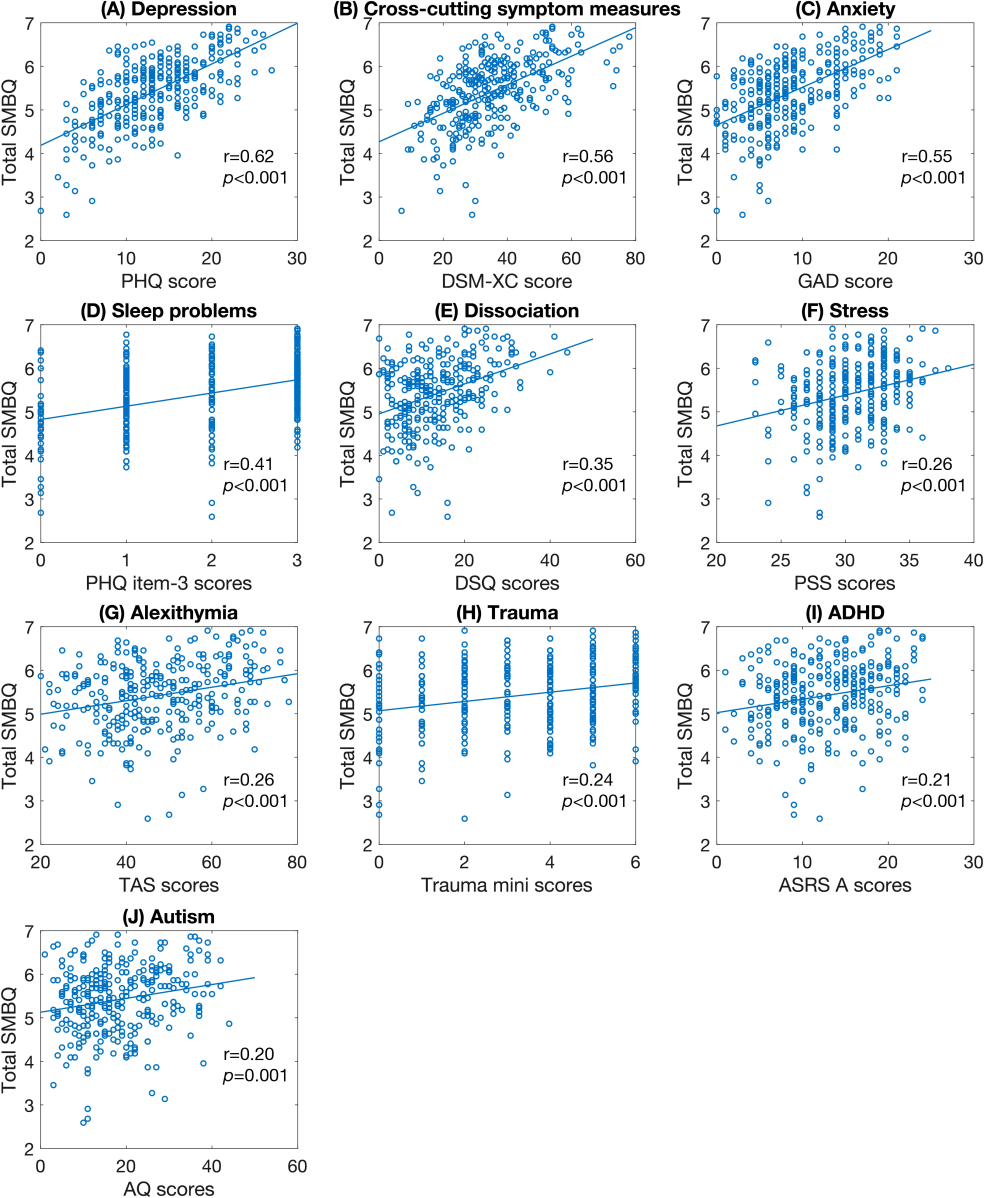
Scatter plots of significant correlations between SMBQ scores and psychiatric factors. **(A)** depression symptoms, **(B)** cross-cutting psychiatric symptoms, **(C)** anxiety symptoms, **(D) **sleep problems, **(E)** dissociative symptoms, **(F)** perceived stress level, **(G)** alexithymic traits, **(H)** number of past traumatic experiences, **(I)** ADHD symptoms, and **(J)** autistic traits. The *p*-values are false discovery rate (FDR) adjusted for multiple comparisons. SMBQ, Shirom-Melamed Burnout Questionnaire; PHQ, Patient Health Questionnaire; DSM-XC, DSM-5 Cross-cutting Symptom Measure; GAD-7, Generalized Anxiety Disorder Scale; DSQ, Dissociation Screening Questionnaire; PSS, Perceived Stress Scale; TAS, Toronto Alexithymia Scale; ADHD, Attention-deficit/Hyperactivity disorder; ASRS, Adult ADHD Self-report Scale; AQ, Autism-spectrum Quotient.

A large number of psychiatric measures were highly correlated with burnout symptoms. Specifically, total SMBQ scores correlated with depression (PHQ scores), cross-cutting measures of mental illness (DSM-XC scores), anxiety (GAD scores), sleep problems (PHQ item-3 scores), dissociation (DSQ scores), stress (PSS scores) and alexithymia (TAS scores) (**Figures 8A-G**), as did all the subscale scores. Experienced traumatic events, symptoms of ADHD (ASRS-A scores) and autistic traits (AQ scores) correlated significantly with total SMBQ (**Figures 8H-J**) and all subscale scores except listlessness.

**Figure 9:**
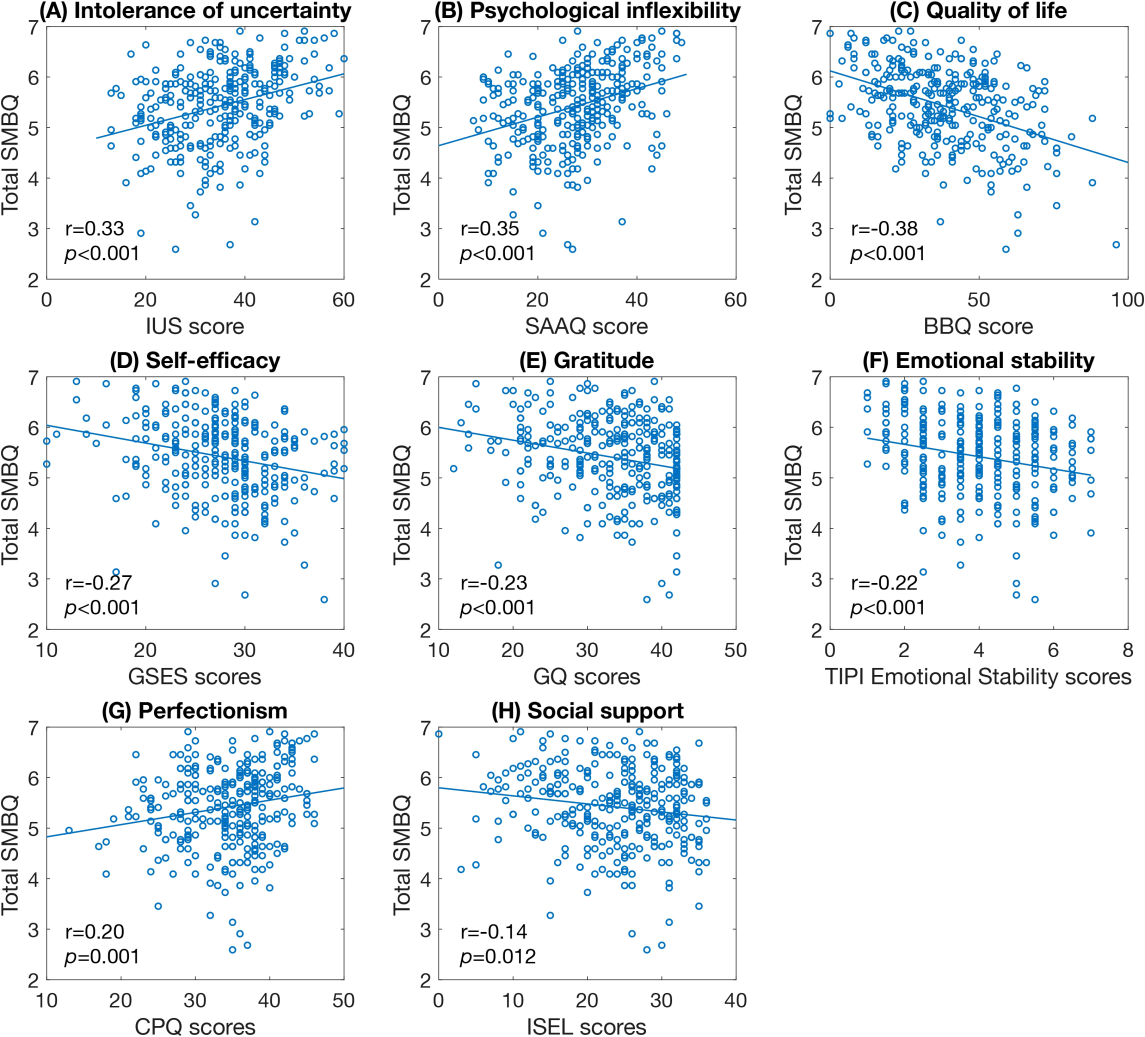
Scatter plots of significant correlations between SMBQ scores and psychological factors. **(A)** intolerance of uncertainty, **(B)** level of psychological inflexibility, **(C)** quality of life, **(D)** perceived self-efficacy, **(E)** proneness to experience gratitude, **(F)** level of emotional stability, **(G)** perfectionism, and **(H)** perceived social support. The *p*-values are false discovery rate (FDR) adjusted for multiple comparisons. SMBQ, Shirom-Melamed Burnout Questionnaire; IUS, Intolerance of Uncertainty Scale; SAAQ, Swedish Acceptance and Action Questionnaire; BBQ, Brunnsviken Brief Quality of Life; GSES, General Self-efficacy Scale; GQ, Gratitude Questionnaire; TIPI, Ten Item Personality Inventory; CPQ, Clinical Perfectionism Questionnaire; ISEL, Interpersonal Support Evaluation List.

Several psychological measures also correlated significantly with burnout symptoms. Total SMBQ scores correlated with intolerance of uncertainty (IUS scores), psychological inflexibility (SAAQ scores), quality of life (BBQ scores), self-efficacy (GSES scores), and gratitude (GQ scores) (**Figures 9A-F**), as did all SMBQ subscale measures. Also, the personality trait emotional stability (TIPI Emotional stability scores) and perfectionism (CPQ scores) correlated with total SMBQ scores (**Figures 9F, G**), physical exhaustion, and tension (**Table 4**). Finally, social support (ISEL scores) correlated significantly with total SMBQ scores (**Figure 9H**) and physical exhaustion.

The total duration of ED sick leave was only significantly correlated with age, sick leave in the past year, and salary level (**Table 5**). Sick leave in the past year was significantly correlated with several measures, including years of schooling, total sick leave, physical demand at work, sleep problems, burnout symptoms including total SMBQ scores, cognitive fatigue and physical exhaustion, cross-cutting measures of mental illness (DSM-XC scores), experienced traumatic events, autistic traits (AQ scores), dissociation (DSQ scores), depression (PHQ scores), quality of life (BBQ scores), and perfectionism (CPQ scores) (**Table 5**).

**Table 5:** Significant correlations with sick leave measures.

Category	Total sick leave		
	Measure	ρ	*p*
*Demographic*	Age	0.22	0.003
*Occupational*	Sick leave past year (%)	0.44	0.000
	Salary level	-0.19	0.015
Category	Sick leave in the past year		
	Measure	ρ	*p*
*Demographic*	Schooling	-0.17	0.015
*Occupational*	Total sick leave	0.44	0.000
	Physical demand at work	0.18	0.015
*Psychiatric*	Sleep problems	0.17	0.015
	SMBQ-Total	0.18	0.015
	SMBQ-Cognitive fatigue	0.16	0.022
	SMBQ-Physical exhaustion	0.15	0.023
	DSM‐XC	0.15	0.027
	Trauma	0.14	0.032
	AQ	0.13	0.043
	DSQ	0.13	0.043
	PHQ	0.13	0.043
*Psychological*	BBQ	-0.17	0.015
	CPQ	0.13	0.043

One-tailed correlation tests with FDR-adjusted *p*-values.

AQ, Autism-spectrum Quotient; ASRS, Adult ADHD Self-report Scale; BBQ, Brunnsviken Brief Quality of Life; BMI, Body mass index; CPQ, Clinical Perfectionism Questionnaire; DSQ, Dissociation Screening Questionnaire; GAD, Generalized Anxiety Disorder Scale; GQ, Gratitude Questionnaire; GSES, General Self-efficacy Scale; HAD, Hospital Anxiety and Depression Scale; ISEL, Interpersonal Support Evaluation List; IUS, Intolerance of Uncertainty Scale; KEDS, Karolinska Exhaustion Disorder Scale; PHQ, Patient Health Questionnaire; PSS, Perceived Stress Scale; SAAQ, Swedish Acceptance and Action Questionnaire; SMBQ, Shirom-Melamed Burnout Questionnaire; TAS, Toronto Alexithymia Scale.

KEDS scores were strongly positively correlated with total SMBQ scores (ρ = 0.76), indicating a substantial overlap between the two measures. The KEDS scores were especially strongly correlated with the physical exhaustion (ρ = 0.76) and cognitive fatigue subscales (ρ = 0.65) of the SMBQ. KEDS scores were also statistically significantly correlated with most of the same factors as total SMBQ scores. Specifically, KEDS was correlated with scores of depression (PHQ scores), cross-cutting measures of mental illness (DSM-XC scores), anxiety (GAD scores), sleep problems, quality of life (BBQ scores), dissociation (DSQ), demand level at home, intolerance of uncertainty (IUS scores), psychological inflexibility (SAAQ scores), perfectionism (CPQ scores), light exercise frequency, self-efficacy (GSES scores), alexithymia (TAS scores), gratitude (GQ), vigorous exercise frequency, emotional stability, autistic traits (AQ scores), snack consumption, sick leave in the past year, ADHD, mental demand at work, and number of children (all adjusted *p*<0.05) (**Table 6**). Trauma, years of schooling, salary level, PSS scores, workplace physical demand level, ISEL scores, and BMI were the only measures which were correlated with total SMBQ but not KEDS score. On the other hand, KEDS scores, but not total SMBQ scores, were significantly correlated with workplace mental demand level and number of children.

**Table 6:** Significant correlations with KEDS scores.

Category	Measure	ρ	*p*
*SMBQ*	Total score	0.76	0.000
	Cognitive weariness	0.65	0.000
	Listlessness	0.51	0.000
	Physical exhaustion	0.76	0.000
	Tension	0.34	0.000
*Demographic*	Household demand level	0.33	0.000
	Number of children	0.17	0.046
*Lifestyle*	Vigorous exercise	-0.27	0.002
	Light exercise	-0.29	0.001
	Snack consumption	0.21	0.018
*Occupational*	Sick leave past year (%)	0.19	0.026
	Mental demand level	0.18	0.040
*Psychiatric*	PHQ	0.61	0.000
	DSM‐XC	0.55	0.000
	GAD	0.54	0.000
	Sleep problems	0.53	0.000
	DSQ	0.40	0.000
	TAS	0.28	0.002
	ASRS-A	0.18	0.035
	AQ	0.25	0.005
*Psychological*	SAAQ	0.32	0.000
	IUS	0.32	0.000
	CPQ	0.31	0.001
	Emotional stability	-0.27	0.002
	GQ	-0.28	0.002
	GSES	-0.29	0.001
	BBQ	-0.47	0.000

One-tailed correlation tests with FDR-adjusted *p*-values.

AQ, Autism-spectrum Quotient; ASRS-A, Adult ADHD Self-report Scale part A; BBQ, Brunnsviken Brief Quality of Life; BMI, Body mass index; CPQ, Clinical Perfectionism Questionnaire; DSQ, Dissociation Screening Questionnaire; GAD, Generalized Anxiety Disorder Scale; GQ, Gratitude Questionnaire; GSES, General Self-efficacy Scale; HAD, Hospital Anxiety and Depression Scale; ISEL, Interpersonal Support Evaluation List; IUS, Intolerance of Uncertainty Scale; KEDS, Karolinska Exhaustion Disorder Scale; PHQ, Patient Health Questionnaire; PSS, Perceived Stress Scale; SAAQ, Swedish Acceptance and Action Questionnaire; SMBQ, Shirom-Melamed Burnout Questionnaire; TAS, Toronto Alexithymia Scale.

## Discussion

We have presented the baseline characterization of the PROMUS cohort, including an analysis of cross-sectional correlations between symptoms of ED and a broad range of relevant factors. We found that the cohort corresponded well with the typical ED population concerning demographic, occupational, psychiatric and psychological measures. The ED sample included participants with an average age of 38, a majority of women, an average SMBQ score of 5.4, and participants who reported high scores on depression, sleep problems, anxiety, stress, cross-cutting measures of mental illness, perfectionism and psychological inflexibility, and low scores on quality of life. We also found that symptom severity and sick leave correlated with a large number of factors, most notably depression, cross-cutting measures of mental illness, anxiety, sleep problems, quality of life, dissociation, psychological inflexibility, intolerance of uncertainty, self-efficacy, stress, alexithymia, trauma, gratitude, years of schooling, the personality trait emotional stability, household demand level, symptoms of ADHD, autistic traits perfectionism, and exercise.

Regarding the psychological characteristics of the cohort, both women and men scored higher in intolerance of uncertainty, perfectionism, psychological inflexibility, and agreeableness compared to non-clinical reference samples, while women scored significantly lower on self-efficacy. We also found that overall symptom severity was associated with intolerance of uncertainty, perfectionism, psychological inflexibility, emotional stability, gratitude and self-efficacy. These associations highlight potential treatment targets, for instance using cognitive behavioral therapy, and confirm recent findings in ED patients, particularly regarding perfectionism, psychological inflexibility (34, 46) and general self-efficacy (85). Notably, psychological inflexibility was among the factors with the strongest positive associations with ED severity and the ED group scored substantially higher on the SAAQ scale than a reference sample. This adds to the qualitative literature describing recurring feelings of detachment and a need for targeting flexibility to facilitate recovery by ED patients (32, 33, 86–88). Similarly strong associations were observed between burnout symptoms and higher levels of intolerance of uncertainty, and the participants in this study scored significantly higher on IUS scores than a general sample. Feelings of uncertainty have been noted in the ED lived experience literature concerning the recovery process, demands and expectations of others and the workplace, and the future itself (32, 86, 89). Intolerance of such uncertainty may conceivably add to the burden of ED, thus increasing the perceived severity of symptoms.

Personality traits have been given much attention in burnout research, with all big five personality traits being linked to burnout symptoms to varying degrees. However, only emotional stability – the inverse of neuroticism – was significantly correlated with ED symptoms in the present study. This is perhaps not too surprising, given that neuroticism is typically the personality trait most strongly correlated with burnout symptoms by far (90), and has also been identified as a key factor in burnout-related exhaustion symptoms specifically (e.g (91–93).). In contrast to previous findings, however, none of the other personality traits were negatively correlated with ED symptoms. Instead, we found that our cohort scored higher than the general population on agreeableness, contradicting previous studies hypothesizing that higher levels of agreeableness may be protective against burnout (75) and confirming a negative association with burnout levels (48, 75). Agreeableness reflects a tendency toward compassion, cooperation, and a prosocial orientation in interpersonal interactions. The elevated levels of agreeableness observed in our cohort may be attributable to the sample’s composition, which is predominantly drawn from caregiving professions (nurses, teachers, social worker etc.). These occupations often attract individuals with high levels of empathy and a strong desire to support others—characteristics closely aligned with high agreeableness. Further research is therefore needed to elucidate the role of personality traits within the ED population, especially compared to a matched control group, and to clarify why our findings diverge from previous results reported in the broader burnout literature.

Psychiatric factors significantly correlated with burnout symptoms included stress, cross-cutting measures of mental illness, anxiety, depression, sleep problems, alexithymia, dissociation, ADHD and autistic traits. These findings reflect the major comorbidities and general psychiatric issues previously observed in ED patients. We also found that burnout symptoms correlate with traumatic experiences, supporting previous findings that trauma may increase the vulnerability of developing burnout symptoms (36). Indeed, early-life adversity is a recurrent characteristic of ED patients (86). For instance, ED diagnosis is associated with a 4 times higher risk of being diagnosed with PTSD compared to the general population (31). Unfortunately, we did not include a specific questionnaire for PTSD symptoms and we did not assess the quantity or severity of traumatic experiences. Further studies are required to better understand the role of trauma in ED. Particularly noteworthy, however, is the large proportion (61%) of participants who reported sexual abuse. This incidence is higher than recently reported in the general population, where the prevalence of sexual harassment experience in the previous 12 months is estimated at 33.4% in women aged 16–24 in Sweden (94). This discrepancy may partially be explained by the broad framing of our prompted question (“Has someone tricked or forced you to perform sexual acts?”, including examples of sexual acts such as sending or receiving genital photographs online), and our sample being substantially older. Further research is therefore required to investigate the role of sexual abuse as a risk factor for developing ED.

Sleep problems emerged as one of the strongest correlations with burnout symptoms, consistent with both the ED diagnostic criteria and the high frequency of sleep disturbances reported in ED by patients (29). There are several mechanisms which may account for this relationship. For instance, poor sleep may contribute to a reduced ability to recover from stress effectively, but increased fatigue may also disrupt circadian patterns hampering patients’ ability to sleep (95). Mediating factors may include reduced physical activity, an increase of which may help promote sleep and reduce perceived stress. Nonetheless, without further mediation analyses, these mechanisms remain speculative.

There was also a correlation between burnout symptoms and alexithymia, confirming previous findings in both the general population (35) and healthcare workers (96). However, only 2% of participants had scores indicative of alexithymia, suggesting that clinically relevant levels of alexithymia were not prevalent in the current sample. Similarly, although only 10% of participants reported a DSQ score above the cutoff for dissociation, we found a significant association between ED severity and symptoms of dissociation. Although dissociation per se is not well researched in the context of persistent stress, patients with ED describe feelings of detachment from the self and the environment (32, 33) in a process similar to depersonalization which is a core feature of burnout. The observed correlation between dissociation and burnout symptoms merits further research.

Notably, 46% of the participants scored above the cut-off indicative of the neurodevelopmental disorder ADHD (although only 10% reported an ADHD diagnosis) and we found significant associations between ED severity and ADHD symptom level. However, the ASRS-A questionnaire includes only six questions, several of which correspond to symptoms of ED fairly well, including questions on memory, concentration and delaying demanding tasks (such as “How often do you have problems remembering appointments or obligations?”), suggesting that this result may reflect symptom overlap rather than increased ADHD traits. Still, a recent large-scale study investigating the prevalence of psychiatric diagnoses following ED found that an ED diagnosis was associated with approximately 4 times higher risk of being diagnosed with ADHD compared to the general population (31). This lends credence to the idea that ADHD may be a predisposing factor increasing the risk of developing ED, possibly by increasing exerted cognitive demand in daily or work-related tasks (97). It is also possible that burnout symptom may worsen pre-existing ADHD-related issues, e.g. with attention and executive functioning. We also found significant associations between exhaustion symptom severity and autistic traits, although not as notable as with ADHD, supporting the finding by Wallensten et al. of an increase in the incidence of autism diagnoses following ED (31). Overall, these findings highlight the importance of addressing neurodevelopmental conditions in the treatment of ED.

We also examined correlations with each SMBQ subscale individually. Interestingly, the correlations for the listlessness subscale diverged from those of the total score, while the remaining subscales —tension, cognitive weariness, and somatic symptoms— showed patterns largely consistent with the total score. Specifically, listlessness did not correlate with household demand level, physical demand at work, autistic traits, or ADHD symptoms—all of which showed significant associations with the total SMBQ score and the other subscales. This pattern suggests that the listlessness subscale may tap into a distinct dimension of exhaustion, potentially reflecting subjective vitality or energy depletion that is less directly influenced by external demands or neurodevelopmental traits. Future research should investigate this divergence further to clarify its theoretical and empirical underpinnings.

Cognitive fatigue was the only measure with a significant correlation with the number of children, which is consistent with reduced cognitive capacity in women without ED who had more children (98) and may also be associated with age (99).

SMBQ, our primary symptom measure, was developed to assess symptoms of burnout and the results may therefore be more relevant to a broader population with chronic stress symptoms. However, we also assessed specific ED symptoms using KEDS in approximately half of the participants (n=130). KEDS scores correlated significantly with the same factors as SMBQ, with a few exceptions: trauma, years of schooling, stress (PSS scores), workplace physical demand level, support (ISEL scores), and BMI. Hence, these specific factors may be less relevant to the symptoms of ED per se; however, the different findings could also be due to the reduced power of the smaller KEDS sample. Additionally, KEDS scores, but not SMBQ scores, correlated significantly with workplace mental demand level and number of children. This may suggest that cognitive load — both at work and in the home environment — could be particularly relevant to the core symptomatology of ED as captured by the KEDS scale, which directly assesses perceived demands in daily life. The number of children may reflect competing demands on attentional resources, rest opportunities, and recovery time, while high mental demands at work may amplify the load on already impaired cognitive functioning. These findings warrant further investigation into how overlapping cognitive demands from work and home environments interact to influence the development and maintenance of ED and burnout.

This study has some notable strengths. First, it is based on a relatively large and clinically well-defined sample, using the diagnostic criteria for ED in the Swedish ICD-10 system. This enhances diagnostic precision and reduces heterogeneity, ensuring that the observed associations are specific to chronic stress-related exhaustion. Second, the use of a broad set of validated self-report questionnaires spanning demographic, occupational, psychiatric, psychological, and lifestyle domains enables a multidimensional analysis of factors associated with symptom severity. This approach lays the groundwork for more refined follow-up studies focusing on the most relevant factors. Third, the dimensional design, analyzing associations within the ED group rather than relying on categorical comparisons with a control group, allows for a more nuanced understanding of individual differences in symptomatology. Fourth, the cohort is linkable to national health and social insurance registries, enabling future longitudinal research on outcomes and trajectories. Finally, the study contributes to the relatively limited international literature on ED (14), addressing an under-recognized yet growing public health concern.

This study also has several limitations. First, sick leave due to ED diagnosis was self-reported and we did not conduct any control to verify the participants’ reports. Hence, there may be participants in the study who actually did not fulfill these criteria. In particular, there may be participants who were on sick leave due to other conditions than the specific ICD-10 diagnosis (F43.8A). However, since this was a dimensional study using the questionnaires as measures, rather than comparing nosological groups, this is not a major shortcoming.

Second, a notable limitation is the exclusive reliance on self-reported questionnaire measures for data collection. While self-reported data provide valuable insights into subjective experiences, they are inherently vulnerable to biases such as social desirability and recall bias. Additionally, the use of multiple self-reported measures introduces the possibility of common method bias, which could inflate the observed correlations between variables due to shared method variance rather than reflecting true relationships between the constructs. To address this concern, we conducted Harman’s single-factor test to assess the presence of common method bias. The results indicated that the variance explained by a single factor was 15%, suggesting that common method bias is unlikely to have significantly influenced the findings of this study. However, despite this reassuring result, the sole use of self-reports limits the generalizability of the findings. Future research would benefit from integrating multi-method approaches, including objective or observational data, to triangulate findings and reduce potential biases inherent in self-report methods. Also, our study utilizes a longitudinal design including brain imaging data, which will be published in separate reports. These data will offer more objective insights into the relationships observed here, allowing for a more comprehensive understanding of the temporal dynamics and neurobiological underpinnings of the studied constructs. The inclusion of imaging data in future analyses will help mitigate the limitations associated with self-report measures and provide a more robust framework for interpreting the findings.

Third, most of the questionnaires used were designed to assess general psychological or behavioral constructs rather than constructs specific to ED. As a result, the measures may lack the specificity required to capture the nuanced ways these constructs manifest in ED. For example, while tools like the PHQ and GAD provide valuable insights into depression and anxiety, they may not fully account for the unique patterns or interactions of these symptoms within the context of ED.

Fourth, all questionnaires were administered electronically rather than in person. While this mode of administration offers practical advantages in terms of convenience and efficiency, it may introduce several limitations. For instance, the absence of direct interaction with researchers may lead to variability in how participants interpret or engage with questionnaire items. More importantly, exclusive reliance on electronic data collection may result in the underrepresentation of certain populations—particularly older adults, individuals with limited digital literacy, those with lower educational attainment, or people living in rural areas with limited internet access. These factors could contribute to selection bias and affect the generalizability of the findings. To enhance sample representativeness and data quality in future research, it will be important to consider complementary methods of data collection, such as paper-based or interviewer-administered formats.

Fifth, we did not collect information about medication, which may impact the results, in particular with regard to the common use of antidepressant and anxiolytic medication in ED or stimulants in ADHD.

Sixth, the generalizability of our findings may be limited by the demographic composition of the sample, which consisted predominantly of women under the age of 50, and all participants were recruited from the Gothenburg area of Sweden. Although these characteristics reflect the typical clinical presentation of ED in Sweden, where the diagnosis is disproportionately observed in working-age women, often employed in caregiving professions. Nevertheless, this demographic homogeneity may restrict the external validity of the results, particularly in relation to male patients, older adults, or populations in other cultural or healthcare contexts. Future studies should aim to replicate these findings in more diverse and representative samples to assess their broader applicability.

Finally, the reported results are cross-sectional only, limiting inference of causality regarding the associations observed. For example, while reduced physical activity was associated with higher symptom severity, it remains unclear whether inactivity contributes to the development of symptoms, or whether it is a consequence of symptom burden. As such, the directionality of these relationships cannot be determined. Longitudinal research is needed to track changes in symptoms and related behavioral or psychological factors over time, and to identify potential causal pathways. In addition, interventional studies—such as those evaluating the effects of exercise programs on symptom outcomes—are warranted to test the efficacy of modifiable targets in the treatment of exhaustion disorder.

With these limitations in mind, this study provides a comprehensive baseline characterization of the PROMUS cohort and identifies a broad range of factors cross-sectionally associated with ED severity. Importantly, however, these results provide the initial stepping stone for future analyses of additional time points that will allow a detailed understanding of the temporal dynamics of these associations, as well as the disentanglement of potential interdependencies and complex relationships using more sophisticated analysis methods such as structural equation modeling. Moreover, the inclusion of brain imaging data promises to support a neurobiological framework for understanding ED.

Although we examined patients with ED, our findings apply to chronic stress conditions more broadly. Specifically, the associations with a broad range of factors align with a biopsychosocial framework, highlighting the interplay of biological, psychological, and social factors in developing and maintaining chronic stress disorders, underscoring the importance of integrative prevention and treatment approaches to alleviate chronic stress and support recovery. As such, our findings support several established treatment methods, such as cognitive behavioral therapy (100) and multimodal intervention (101), but also raise the promise of novel strategies such as psychedelic-assisted therapy which is effective in treating symptoms of depression and increasing psychological flexibility (102–104). In light of significant correlates such as low physical activity, high psychological demands, and reduced psychological flexibility, our results also point to concrete targets for public health interventions—including workplace-based stress management programs, community-level exercise promotion, and support for work-life balance. However, investigating biological factors, including brain imaging, is essential for comprehensively understanding the underlying mechanisms of these conditions and identifying pathways for potential change.

In sum, the findings from this study underscore the multifaceted nature of ED in particular, and chronic stress in general, and highlight the significant role of demographic, psychiatric, psychological, occupational, and lifestyle factors in its manifestation. In particular, the results highlight the high symptom burden described by patients, that overlap across a spectrum of psychiatric diagnoses.

## Data Availability

The raw data supporting the conclusions of this article will be made available by the authors, without undue reservation.

## Supplementary material

Supplementary Table 1 Correlations with SMBQ scores.

Supplementary Table 2 Correlations with sick leave measures.

Supplementary Table 3 Correlations with Karolinska exhaustion disorder scale (KEDS) scores.
